# Determination of Selected Pesticide Residues in Wheat Flour by GC–MS

**DOI:** 10.3390/molecules31203726

**Published:** 2026-10-17

**Authors:** Antonella Maria Aresta, Nicoletta De Vietro, Carlo Zambonin

**Affiliations:** Department of Biosciences, Biotechnology and Environment, University of Bari “Aldo Moro”, Via Edoardo Orabona 4, 70125 Bari, Italy

**Keywords:** pesticide residues, organophosphorus pesticides, organochlorine pesticides, GC–MS, ion-trap mass spectrometry, wheat flour, food safety

## Abstract

The widespread use of pesticides in modern agriculture contributes to a range of environmental and food-safety concerns, including the persistence of chemical residues in food products, contamination of soil and water, and potential risks to human and ecosystem health. Among food and feed commodities, cereal-based products represent an important potential source of dietary exposure due to their extensive consumption and the possibility of residue transfer from wheat grains to processed flours. In this study, a direct acetone extraction combined with gas chromatography–ion-trap mass spectrometry (GC–MS) was explored as a simplified analytical approach for the determination of selected pesticide residues in wheat flour. Nine pesticides were initially screened, and six compounds (ethoprophos, dimethoate, terbufos, diazinon, chlorpyrifos, and methoxychlor) were retained for method development based on their extractability and chromatographic behavior. Chromatographic and ionization conditions were optimized in Full Scan, SIM, and MS/MS modes, and matrix-matched calibration was applied to compensate for matrix-induced signal variations. The method was evaluated in a composite wheat-flour matrix, demonstrating acceptable process efficiency, a characterized matrix effect, and satisfactory quantitative performance for four analytes over the experimentally evaluated range of 5–50 µg/kg. Two commercial flour samples contained estimated diazinon concentrations of 0.4 and 1.0 µg/kg, respectively, which were further assessed using the standard addition method. Overall, this study provides a proof of concept for the use of direct acetone extraction coupled with ion-trap GC–MS for the exploratory monitoring of selected pesticide residues in wheat flour. Full method validation in accordance with SANTE/11312/2021 v2026 was outside the scope of this work.

## 1. Introduction

Pesticides, also referred to as plant protection products, encompass a wide range of chemical compounds used to protect crops against insects, fungi, weeds, and other harmful organisms. Although their application has substantially improved agricultural productivity, pesticides also represent a potential source of contamination of soil, surface water, groundwater, and food intended for human consumption [1,2,3]. Among the principal classes of analytical and toxicological interest are organophosphorus pesticides (e.g., dimethoate, diazinon, and chlorpyrifos), which inhibit acetylcholinesterase activity; organochlorine pesticides (e.g., aldrin and methoxychlor), characterized by high environmental persistence and bioaccumulation potential; and herbicides, such as bentazone. Owing to their structural diversity, these compounds exhibit markedly different environmental behaviors, including variations in mobility, lipophilicity, volatility, and degradation pathways [4].

Wheat is one of the most extensively cultivated cereal crops worldwide and represents a major source of nutrients for both humans and livestock [5]. It provides dietary fibre, carbohydrates, proteins, vitamins, and essential micronutrients [6]. Wheat-derived products, including bread, pasta, baked goods, and other cereal-based foods, account for a substantial proportion of daily caloric intake and are consumed by all population groups, including children and women of reproductive age, who are particularly susceptible to toxicological risks associated with chemical contaminants [7,8,9].

For decades, synthetic pesticides have played a fundamental role in crop protection and grain preservation during storage, becoming an integral component of modern agricultural production. Nevertheless, both pre- and post-harvest applications may result in the persistence of pesticide residues in food and feed commodities. Furthermore, the use of insecticides, fungicides, and rodenticides during grain transport and storage may further increase residue levels. Milling and processing operations can also influence residue distribution because pesticides tend to partition into specific grain fractions according to their physicochemical properties, particularly polarity and lipophilicity [10]. Increasing evidence demonstrates that pesticide residues may adversely affect ecosystems and human health, raising significant environmental and public health concerns [11,12]. Consequently, integrated pest management (IPM) strategies have been promoted within the European Green Deal, which—through the EU Farm to Fork Strategy and the EU Biodiversity Strategy for 2030—sets the objective of reducing the use and risk of chemical pesticides by 50% by 2030 [13].

Considering the central role of wheat and wheat-derived products in human and animal nutrition, continuous monitoring throughout the cereal production chain is essential. Even trace concentrations of pesticide residues may contribute to cumulative dietary exposure, an issue receiving increasing attention from regulatory authorities. Within the European Union, pesticide residues are regulated through a comprehensive legislative framework. Regulation (EC) No. 396/2005 establishes maximum residue levels (MRLs) for food and feed, which are periodically updated [14], whereas Regulation (EC) No. 1107/2009 governs the approval of active substances used in plant protection products [15]. In Italy, pesticide residues in plant foods are monitored through regional and national food-control programs, whereas the National Residue Plan (PNR) specifically covers veterinary drug residues and other contaminants in food of animal origin [16].

In recent years, the use of highly hazardous pesticides has progressively declined. A notable example is chlorpyrifos and chlorpyrifos-methyl, whose approvals were not renewed in the European Union in 2020 following evidence of developmental neurotoxicity and potential endocrine-disrupting effects [17].

Against this background, the determination of pesticide residues in flour is essential to ensure food safety and regulatory compliance. Consequently, there is growing interest in simple, rapid, and cost-effective sample preparation procedures coupled with highly selective instrumental techniques for pesticide residue analysis in cereal products.

The QuEChERS (Quick, Easy, Cheap, Effective, Rugged, and Safe) method is currently regarded as the reference extraction procedure for multi-residue pesticide analysis in food matrices. The method involves extraction with acetonitrile, followed by salting-out partitioning and dispersive solid-phase extraction (d-SPE) clean-up prior to GC–MS/MS or LC–MS/MS analysis [18,19]. Originally developed for high-moisture, low-fat matrices, QuEChERS has subsequently been adapted for dry and high-fat commodities by incorporating water addition and modified clean-up procedures [20,21,22,23].

Although QuEChERS enables the simultaneous determination of hundreds of pesticides, its application to wheat flour remains challenging because of the variable starch, protein, lipid, and moisture contents of different flour types [22]. These characteristics frequently require additional sample preparation steps, including prolonged extraction, hydration, freezing clean-up, and extensive purification, thereby increasing analytical complexity and partially offsetting the original simplicity of the method. For this reason, alternative sample preparation strategies have also been investigated. Among these, headspace solid-phase microextraction (HS-SPME) has attracted considerable interest because it is solvent-free, minimizes sample handling, and can improve analytical sensitivity for volatile compounds [24]. However, HS-SPME exhibits significant limitations for pesticide residue analysis. Most non-volatile and highly polar pesticides display poor partitioning into the headspace and limited affinity for conventional fiber coatings, resulting in low extraction efficiencies. Moreover, fiber saturation and limited sorption capacity may compromise quantitative performance in complex matrices such as wheat flour. Current research is therefore focused on the development of novel fiber coatings and optimized extraction conditions to expand analyte coverage and improve method robustness [25].

The nine pesticides included in this work were selected to cover substances historically or currently associated with wheat cultivation and storage, including approved compounds, non-renewed active substances of regulatory interest, and legacy contaminants occasionally reported in cereal commodities. Their selection reflects EU residue definitions and MRL relevance [14,15], as well as monitoring priorities for both current-use and obsolete pesticides in wheat-based food chains [16,17].

In the present study, a simplified GC–MS method based on direct acetone extraction and ion-trap mass spectrometric detection was developed and validated for the determination of selected organophosphorus, organochlorine, benzothiadiazine, and triazole pesticide residues in wheat flour. Unlike conventional QuEChERS-based approaches, the proposed procedure was specifically designed for the targeted determination of a limited number of pesticides, reducing sample preparation time, minimizing solvent consumption and matrix manipulation, and providing a robust, cost-effective analytical method suitable for routine laboratories equipped with single-stage GC–MS instrumentation.

## 2. Results

### 2.1. GC–MS Method Optimization

The GC–MS–EI–IT method was optimized to achieve baseline separation of the nine target pesticides within 26 min. Figure 1 shows the chromatogram of a mixed standard solution (1 μg/mL) acquired in Full Scan mode together with the corresponding EI mass spectra, which were consistent with those reported in the NIST library [26].

Following optimization of the chromatographic, acquisition, and identification conditions reported in the Materials and Methods (Section 4.4), the instrumental method was validated according to the criteria described in Section 4.5.

### 2.2. Validation of the Instrumental Method

The analytical performance of the GC–MS method was assessed by evaluating linearity range, regression equation, correlation coefficient (R^2^), and limit of detection (LOD) and limit of quantification (LOQ) for each analyte in SIM and MS/MS acquisition modes. The results are summarized in Table 1.

Excellent linearity was obtained for all analytes, with correlation coefficients (R^2^) ranging from 0.9988 to 0.9999. LODs ranged between 0.02 and 1.0 ng/mL, while LOQs ranged between 0.08 and 3.3 ng/mL, and varied depending on the mode of acquisition for the same analyte. As LOQ was estimated mathematically using the SD/slope approach, its value may be slightly lower or higher than the first calibration point. The latter represents the lowest concentration experimentally meeting the accuracy (≤20% RE) and precision (≤20% RSD) criteria and therefore corresponds to the lowest validated level.

### 2.3. Optimization of Sample Extraction

The performance of the optimized GC–MS method was subsequently evaluated in flour matrices. As described in Section 4.2, different commercial wheat flours were selected for this study, representative of the flour types included in the composite matrix used for method development, including durum wheat flours, soft wheat flours, and blends of both. To account for the compositional variability among wheat flours, a composite sample was prepared by mixing equal portions of the selected flour types. The extraction performance of four solvents (acetone, methanol, dichloromethane, and ethyl acetate) was evaluated using composite flour samples fortified at 20 μg/kg, according to the procedure described in Section 4.2. Among the solvents evaluated, none was able to efficiently extract all the selected pesticides. Preliminary recovery tests indicated that methanol, dichloromethane, and ethyl acetate provided insufficient or highly variable recoveries, whereas acetone yielded acceptable extraction performance for chlorpyrifos, diazinon, dimethoate, ethoprophos, methoxychlor, and terbufos. For this reason, only acetone was selected for subsequent method validation. Additional tests showed that adding water to the flour prior to extraction did not improve recovery, confirming that acetone alone was sufficient to efficiently extract the analytes from the dry matrix.

Figure 2 shows the GC-MS-EI-IT chromatograms acquired in SIM and MS/MS relating to composite flour sample added to a concentration of 20 µg/kg.

### 2.4. Validation in Composite Sample

The absence of interfering peaks at the retention times of the target analytes was verified by repeated independent analysis (n = 6) of blank composite flour extracts, according to the specificity assessment procedure described in Section 4.5.7. In an exploratory preliminary assessment, two of the twelve commercial flours were excluded from the composite to obtain a matrix free of detectable residue and therefore suitable for use as a true blank in validation experiments.

Once specificity had been verified, the method was validated in terms of recovery and precision. Table 2 summarizes the results obtained for the six selected pesticides in the composite flour matrix at the three fortification levels (5, 20, and 50 μg/kg), according to the procedure described in Section 4.5.2.

Among the six investigated analytes, dimethoate showed markedly low process efficiency (13–30%), whereas chlorpyrifos exhibited lower values (64–68%) and comparatively high variability. These results indicate limitations in the overall analytical performance for these two compounds, particularly for dimethoate. Accordingly, the extraction procedure was considered suitable for the other four analytes, while its applicability to dimethoate and chlorpyrifos remains limited under the conditions investigated [21,27].

Prior to evaluating the analytical performance of the method, the stability of fortified flour extracts was assessed according to the procedure described in Section 4.5.6. The chromatographic responses obtained after storage at 4 °C for 7 days and at room temperature for 24 h were consistent with those measured immediately after preparation. No evidence of degradation, abnormal drift, or retention-time shifts was observed over the tested periods.

After determining extraction performance at three fortification levels, matrix-matched calibration curves were constructed by spiking the composite flour at five concentration levels before extraction.

Matrix-matched calibration showed excellent linearity for all analytes (R^2^ = 0.9978–0.9999). The corresponding calibration equations, analytical sensitivities, LODs, and LOQs are summarized in Table 3. LOQs were estimated using the SD/slope approach and represent instrumental detection capability. The lowest calibration point corresponds to the lowest concentration experimentally meeting accuracy and precision criteria.

### 2.5. Analysis of Commercial Samples

The applicability of the validated method was evaluated by analyzing twelve commercial flour samples in SIM mode. Diazinon residues were detected in two samples, namely one durum wheat semolina and one type 00 soft wheat flour. Representative MS/MS chromatograms of the contaminated samples and the corresponding blanks are shown in Figure 3.

As the limits estimated in Table 3 cannot be considered experimentally validated reporting limits, because method performance at these concentration levels was not established through fortification experiments (the lowest experimentally evaluated fortification level was 5 μg/kg), the estimated diazinon concentrations (0.4 and 1.0 μg/kg) were further confirmed by the standard addition method. Standard additions corresponding to 1-, 2-, and 5-fold the estimated residue concentration were performed on each naturally contaminated sample according to the procedure described in Section 4.5.1. The concentrations obtained by standard addition agreed well with those determined by matrix-matched calibration. This agreement provided supplementary support for the estimated concentrations, although it did not establish quantitative performance at these levels.

## 3. Discussion

### 3.1. Chromatographic and Acquisition Optimization

Peak broadening, peak distortion, and carry-over are common issues in GC analysis and can substantially compromise chromatographic performance, particularly in the determination of pesticide residues at trace levels [28]. These phenomena are often exacerbated by matrix constituents and by interactions between analytes and active sites within the injector system. Therefore, particular attention was devoted to optimizing the injection conditions. The use of an acetone: isooctane (80:20, *v*/*v*) injection solvent proved to be an effective compromise between analyte solubility and chromatographic performance. Isooctane, a weak and non-polar solvent, reduced solvent expansion during injection and improved analyte transfer onto the analytical column, thereby minimizing peak broadening and carry-over. Similar improvements have previously been reported for thermolabile and semi-volatile pesticide residues analysed by GC–MS [28].

The choice of the injector liner also proved to be a critical factor in achieving satisfactory chromatographic performance. The gold-plated PTV inlet liner equipped with three baffles provided the most reproducible chromatographic responses. The gold-deactivated surface minimized analyte adsorption and catalytic degradation within the injector, whereas the three-baffle configuration promoted efficient sample vaporization and homogeneous transfer of analytes onto the analytical column, resulting in improved peak symmetry and repeatability [29].

Method robustness was evaluated according to the procedure described in Section 4.5.5. Small deliberate variations in carrier-gas flow rate (±0.1 mL min^−1^), injector temperature (±10 °C), and initial oven temperature (±5 °C) did not significantly affect analyte retention times or chromatographic performance, demonstrating the robustness of the optimized chromatographic conditions and supporting the suitability of the method for routine application. Finally, the stability of the standard solutions confirmed the suitability of the selected solvent system for routine analyses. No significant degradation was observed after storage for seven days at 4 °C or for 24 h at room temperature, supporting the practical applicability of the analytical procedure.

As shown in Table 1, both SIM and MS/MS acquisition modes provided excellent linearity (R^2^ > 0.999) for all investigated pesticides, confirming the suitability of both acquisition strategies for quantitative analysis. However, differences in analytical sensitivity were observed depending on the analyte. MS^2^ improved the LOD and LOQ for ethoprophos, terbufos, aldrin and chlorpyrifos, whereas SIM afforded lower detection limits for dimethoate, diazinon and paclobutrazol. Comparable performances were obtained for bentazone and methoxychlor. These differences can be explained by the fragmentation behaviour of the individual compounds in the ion-trap mass spectrometer. In MS^2^ mode, precursor-ion isolation followed by collision-induced dissociation increases analytical selectivity but inevitably reduces the number of ions available for detection. Consequently, improvements in sensitivity are achieved only when fragmentation generates abundant and reproducible product ions [30].

Overall, SIM represented the most versatile acquisition mode for routine quantitative analysis because it provided consistently low detection limits for all target analytes, while MS^2^, on the other hand, offered higher selectivity and proved particularly useful for confirmatory analysis whenever additional confidence in compound identification was required.

### 3.2. Extraction Strategy

Commercial wheat flours exhibit considerable compositional variability, particularly with respect to protein, starch, lipid, moisture, and ash contents, depending on wheat cultivar and degree of milling. These differences may influence analyte extraction, the co-extraction of endogenous compounds, and consequently matrix effects during GC–MS analysis. To account for this variability, a composite flour matrix was prepared by mixing equal portions of different commercial flour types. This approach provided a practical and broadly applicable matrix for method optimization and validation than the use of a single flour type, better reflecting the diversity of samples encountered in routine monitoring.

None of the investigated solvents enabled satisfactory extraction of all the selected pesticides, reflecting the broad range of physicochemical properties covered by the target analytes. Consequently, solvent selection was based on overall analytical performance rather than on the recovery of individual compounds. Acetone was selected for subsequent validation because it provided satisfactory recoveries for chlorpyrifos, diazinon, dimethoate, ethoprophos, methoxychlor, and terbufos, while maintaining full compatibility with direct GC injection and a simplified sample preparation procedure. Matrix-matched calibration was used to compensate for matrix-induced response in the composite blank matrix.

As shown in Table 2, the proposed extraction procedure provided consistent process efficiency (“Before” values) and matrix-induced response (“After” values) across the investigated fortification levels. “Before” values represent overall process efficiency (extraction + matrix effect + instrumental response), whereas “After” values reflect matrix effect and instrumental response only, as both are quantified against solvent standards. Dimethoate and chlorpyrifos showed lower process efficiency, consistent with their known susceptibility to extraction losses and matrix interactions in cereal-based matrices. Both repeatability and within-laboratory reproducibility remained within the recommended precision limits (≤20% RSD), confirming the robustness of the analytical procedure.

Although QuEChERS is widely recognized as the reference sample preparation procedure for multi-residue pesticide analysis in food matrices [18,19,31], its analytical workflow is designed to maximize analyte coverage and therefore includes several extraction and clean-up steps. In contrast, the objective of the present study was the development of a targeted GC–MS method for a limited number of representative pesticides. Under these conditions, a direct solvent extraction strategy was considered more appropriate because it minimizes sample handling, eliminates the need for dispersive clean-up, and produces extracts directly compatible with instrumental analysis. Recoveries comparable to those obtained in the present study have been reported for cereal matrices using QuEChERS followed by GC–MS/MS [19,31].

The exclusion of aldrin, bentazone, and paclobutrazol from matrix validation was attributable to their physicochemical characteristics. Aldrin, due to its pronounced lipophilicity, showed strong interactions with the lipid fraction of the flour matrix. Bentazone, a weakly acidic and highly polar herbicide, exhibited poor extractability under the selected conditions. Paclobutrazol displayed intermediate behaviour, with recoveries likely affected by both moderate lipophilicity and interactions with matrix components. These findings highlight the intrinsic difficulty of developing a single extraction procedure capable of efficiently recovering compounds spanning a wide polarity range.

Overall, the results demonstrate that the proposed extraction strategy is well suited to four of the six pesticides successfully validated in flour and is fully consistent with the goal of developing a rapid, simple and reliable analytical procedure for routine GC–MS monitoring of these residues in flour. The method is therefore appropriate for exploratory applications within the specific matrix and analyte set investigated.

### 3.3. Analytical Performance in Flour Matrix

The excellent linearity achieved using matrix-matched calibration (Table 3) demonstrates the suitability for quantitative analysis in the validated composite wheat-flour matrix, where co-extracted matrix components may influence the GC–MS response. The proposed method exhibited satisfactory analytical sensitivity, with LOQs ranging from 0.1 to 5 μg/kg. Dimethoate showed the highest LOQ (5 μg/kg), which is consistent with its relatively high polarity and lower response under GC–EI conditions compared with the other target analytes. Nevertheless, this value compares favorably with previously reported GC-based methods employing QuEChERS extraction, where a LOQ of 9.3 μg/kg was reported for dimethoate in fruit and vegetable matrices [32]. Even higher LOQs (33 μg/kg) have been reported for cereal matrices [33]. Although direct comparisons should be interpreted with caution because of differences in sample preparation, instrumentation, and validation protocols, these findings demonstrate that the simplified extraction procedure provides analytical sensitivity comparable to previously published GC-based methods within the validated composite blank matrix and, for dimethoate, even lower LOQs.

### 3.4. Applicability of the Method

The detection of diazinon in two commercial flour samples illustrates the potential applicability of the proposed method to the analysis of real cereal products. Although only a limited number of samples was investigated, these findings highlight the relevance of routine monitoring throughout the cereal production chain. The estimated diazinon concentrations (0.4 and 1.0 μg/kg) were considered in the context of current EU pesticide-MRL legislation. Diazinon MRLs were retrieved from the EU Pesticide MRL Database (commodity code 0500090; residue definition: diazinon; Regulation (EC) No 396/2005 and amendments [34]; accessed on 19 August 2026). The current MRL for wheat is 0.01 mg/kg. As wheat flour is a processed product, Article 20 of Regulation (EC) No 396/2005 was considered. In the absence of a validated processing factor for diazinon in cereal milling, a processing factor (PF) of 1 was adopted solely as an illustrative assumption and should not be interpreted as an experimentally established or legally prescribed value. Since the estimated diazinon concentrations were below the lowest experimentally evaluated fortification level, the results were interpreted for exploratory purposes only. The comparison with the wheat MRL was intended to provide regulatory context and does not constitute a compliance assessment for wheat flour. Measurement uncertainty was not quantitatively assessed; therefore, no conclusions regarding regulatory compliance were drawn from these estimates.

## 4. Materials and Methods

### 4.1. Preparation of Standard Solution

Pesticide reference standards (aldrin, bentazone, chlorpyrifos, diazinon, dimethoate, ethoprophos, methoxychlor, paclobutrazol and terbufos) were purchased from Merck (Milano, Italy), and had purity ≥98%. Stock solutions (1 mg/mL) were prepared in acetone and stored at 8 °C. Working solutions were freshly diluted in acetone: isooctane (80:20, *v*/*v*) immediately prior to use. All solvents (acetone, isooctane, methanol, dichloromethane and ethyl acetate) were Pestanal-grade and supplied by Merck. Before analysis, all solutions were filtered through Millipore HV membranes (0.45 μm, Merck) to remove particulates and ensure compatibility with GC-MS injection.

### 4.2. Sample Collection and Preparation

Flour samples were obtained from local suppliers and included three durum wheat, three soft wheat, and six blends of durum and soft wheat flours. To ensure representativeness, a composite sample was prepared by homogenizing equal portions of the selected flours and stored in a dry, moisture-free environment at room temperature until analysis. In addition, for validation purposes, a separate blank composite flour was prepared by pooling only the ten samples that tested negative for all target analytes, to obtain a composite blank matrix, i.e., a sample free of detectable residues. Sample preparation was conducted as follows: 2 mL of acetone was added to 0.5 g of flour. After intensive mixing for 15 min, the sample was centrifuged at 4000 rpm (≈ 2300 × g) for 5 min. The supernatant was filtered through Millipore HV membranes (0.45 μm, Merck) and where no loss of analyte was observed during filtration were then evaporated dry using a Christ RVC 2–18 dryer (Martin Christ Gefriertrocknungsanlagen GmbH, Osterode/Harz, Germany) at room temperature. The residue was redissolved in 200 μL of isooctane: acetone (80:20, *v*/*v*) before analysis. Fortified samples were allowed to equilibrate for 12 h at room temperature, along with the blank samples. Procedural blanks, matrix blanks, and fortified QC samples were included in each batch.

### 4.3. Equipment

A GC–MS Thermo Finnigan Trace Ultra system coupled to a quadrupole ion trap Polaris Q mass spectrometer (Thermo Fisher Scientific, Monza, Italy) was used. Chromatographic separation was achieved using an SPB 5 capillary column (30 m × 0.25 mm × 0.25 µm) (Merck). Helium (99.999%, Nippon Gases, Bari, Italy), was used as the carrier gas at a constant flow of 1.0 mL/min. The PTV injector operated at 250 °C in splitless mode (purge time 1.0 min), and was equipped with a Gold GC inlet liner with three baffles (Thermo Scientific). An injection volume of 1 μL was applied. The oven program was 60 °C (3 min); 10 °C/min to 270 °C; 20 min hold. The transfer line was 250 °C. The ionization source operated in 70 eV Electron Impact (EI) mode, with a source temperature of 250 °C. Data acquisition was managed using Xcalibur 2.0 software (Thermo Fisher).

### 4.4. Acquisition and Identification

Full Scan acquisition (50–450 m/z) was used for spectral library matching (NIST 23, match ≥ 90%). SIM mode targeted the diagnostic ions shown in Table 4.

Structural confirmation was carried out in MS/MS mode using helium as collision gas (1.0–1.5 mTorr). For each analyte, the corresponding precursor–product ion transitions were monitored within the same retention-time windows used for SIM acquisition. Table 5 reports the selected transitions and the associated mass ranges, which were chosen based on the most stable and representative fragments observed in EI spectra and confirmed through comparison with NIST library data.

### 4.5. Method Validation

#### 4.5.1. Linearity

Linearity was evaluated with external curves and standard additions at five levels. In matrix matched curves a weighted regression (1/x) was used following SANTE guidelines (SANTE, 11312, 2021 v2026).

The lowest calibration level was selected as the lowest concentration meeting acceptable accuracy and precision criteria (≤20% RSD and ≤20% RE), and therefore does not necessarily coincide with the mathematically estimated LOQ. Calibration stability was verified through bracketing standards injected at the beginning and end of each analytical batch. Carry-over was assessed by injecting a blank immediately after the highest calibration level, and no detectable peaks were observed at the retention times or diagnostic ions of the target analytes. Samples exceeding the calibrated range were diluted and re-injected to ensure quantification within the validated calibration interval.

To further assess the estimated diazinon concentrations in naturally contaminated flour samples, standard additions were performed at levels corresponding to 1, 2, and 5 times the initially estimated diazinon concentration. Each addition level was evaluated using three independently prepared replicates.

#### 4.5.2. Limits of Detection (LOD) and Quantification (LOQ)

LOD and LOQ were estimated from matrix-matched calibration curves using the standard deviation of replicate measurements in spiked composite flour and the slope of the corresponding calibration curve:

- LOD = 3 × (SD of spiked composite flour matrix)/slope.
- LOQ = 10 × (SD of spiked composite flour matrix)/slope.

For SD determination, six replicate aliquots of composite flour matrix were spiked at levels consistent with predicted LOQs. Chromatographic S/N ratios were also evaluated at the same levels to verify the consistency of these estimates.

The resulting LOD and LOQ values are mathematical estimates based on the variability observed in the fortified flour matrix. They are distinct from the lowest calibration level meeting the predefined accuracy and precision criteria and should not be interpreted as experimentally validated LODs or LOQs.

#### 4.5.3. Accuracy and Recovery

Accuracy was assessed at three representative levels (5, 20 and 50 µg/kg) by recovery assessments. Aliquots were spiked before and after extraction to comply with SANTE criteria to evaluate the overall process efficiency and to isolate the matrix effect and instrumental response, respectively. For each level, six independent replications were performed, following the entire extraction and analytical procedure. The percentage recovery was calculated by comparing the signal of the fortified extracts with that of the solvent standards at the appropriate concentrations (12.5, 50, 125 ng/mL).

#### 4.5.4. Precision

Intra-day accuracy was assessed by analysing six replicates of the same fortified level (5 μg/kg) on the same day. Inter-day accuracy was assessed by repeating the analyses on five different days using a novel spiked composite flour matrix. Results were expressed as a percentage of relative standard deviation (RSD%).

#### 4.5.5. Robustness

Robustness was assessed by introducing controlled variations in the key GC–MS operating parameters, including injector temperature (±10 °C), carrier gas flow (±0.1 mL/min), and initial oven temperature (±5 °C).

The impact of these changes on chromatographic performance (peak area, peak height, and retention-time stability) was assessed to verify that no critical deviations occurred under realistic operating conditions. No significant changes in retention time, ion-ratio behavior, or chromatographic response were observed, supporting the practical robustness of the method.

#### 4.5.6. Stability

The stability of the standards and fortified samples was investigated by storing aliquots of the solutions: at 4 °C for 7 days, and at room temperature for 24 h (operational stability on the bench).

The solutions were re-analyzed at different times, and their chromatographic responses (peak area and retention time) were compared with those obtained immediately after preparation.

#### 4.5.7. Specificity and Selectivity

Specificity was assessed by repeated analysis (n = 6) of unfortified flour extracts (blank samples). Chromatograms acquired in Full Scan, SIM, and MS/MS^2^ modes were inspected at the retention times and diagnostic ions of the target analytes to verify the absence of interfering peaks. No detectable signals were observed at the monitored retention-time windows or ion transitions. Analyte identity in fortified samples was confirmed by matching retention time and mass-spectral features with those of reference standards.

## 5. Conclusions

The GC–MS method described enables the determination of selected pesticides in flour, providing quantitative performance at the experimentally evaluated levels for four of the six investigated compounds, and offering a practical and selective approach for exploratory monitoring in food safety applications. The markedly low process efficiency observed for dimethoate and the lower process efficiency and greater variability observed for chlorpyrifos limit the applicability of the method to these two compounds under the conditions investigated. Despite the technological evolution toward more advanced systems, the ion trap remains a valid analytical solution for preliminary studies and for laboratories with limited instrumental resources. The proposed methodology offers a practical and selective alternative to complex multi-residue approaches, providing a cost-effective tool for food safety surveillance and control of pesticide residues in wheat flour. Future developments may focus on coupling this approach with high-resolution systems (HRMS) and LC–MS/MS to broaden the analytical spectrum and improve the characterization of secondary metabolites, in line with recent EFSA directives on combined contaminant monitoring [14,34].

## Figures and Tables

**Figure 1:**
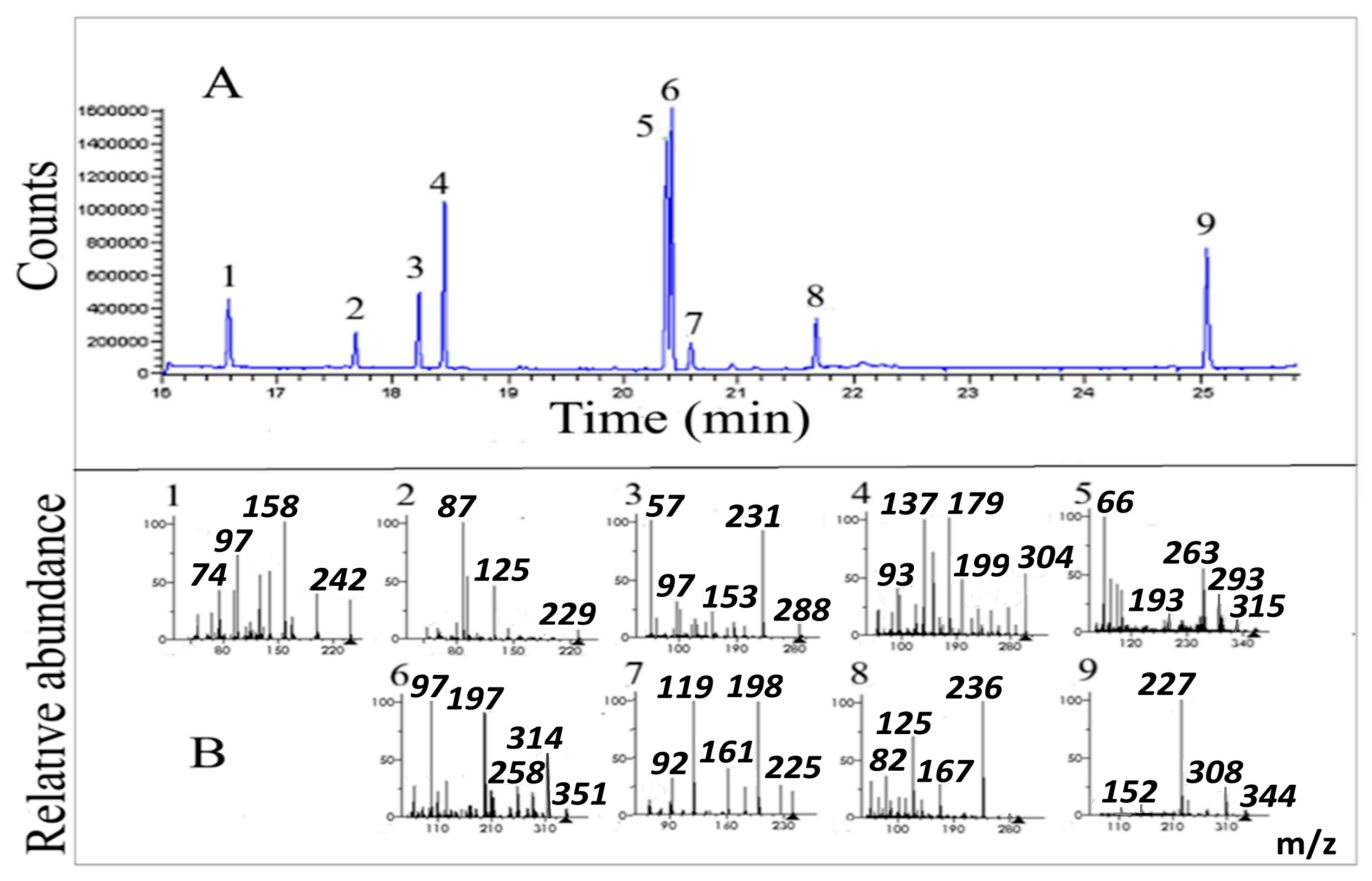
(**A**) GC–MS–EI–IT chromatogram of a mixed pesticide standard solution (1 μg/mL) acquired in Full Scan mode. (**B**) Electron impact (EI) mass spectra of the target analytes: (1) ethoprophos, (2) dimethoate, (3) terbufos, (4) diazinon, (5) aldrin, (6) chlorpyrifos, (7) bentazone, (8) paclobutrazol, and (9) methoxychlor. Experimental chromatographic and mass spectrometric conditions are described in the Section 4.

**Figure 2:**
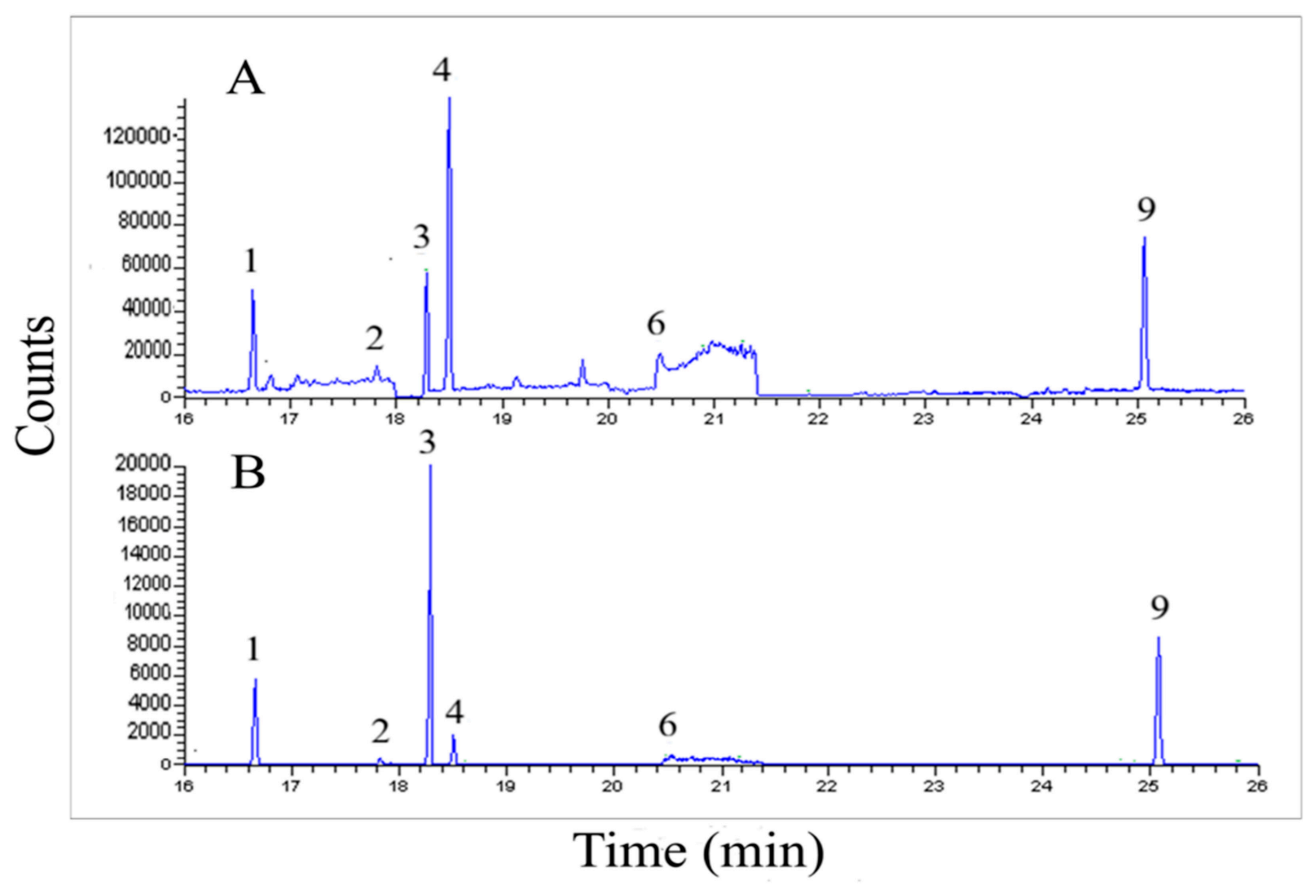
Representative GC–MS chromatograms of a composite flour sample fortified at 20 μg/kg and extracted with acetone, acquired in (**A**) SIM and (**B**) MS^2^ modes. Peaks: (1) ethoprophos, (2) dimethoate, (3) terbufos, (4) diazinon, (6) chlorpyrifos, and (9) methoxychlor. Experimental chromatographic and mass spectrometric conditions are described in the Section 4.

**Figure 3:**
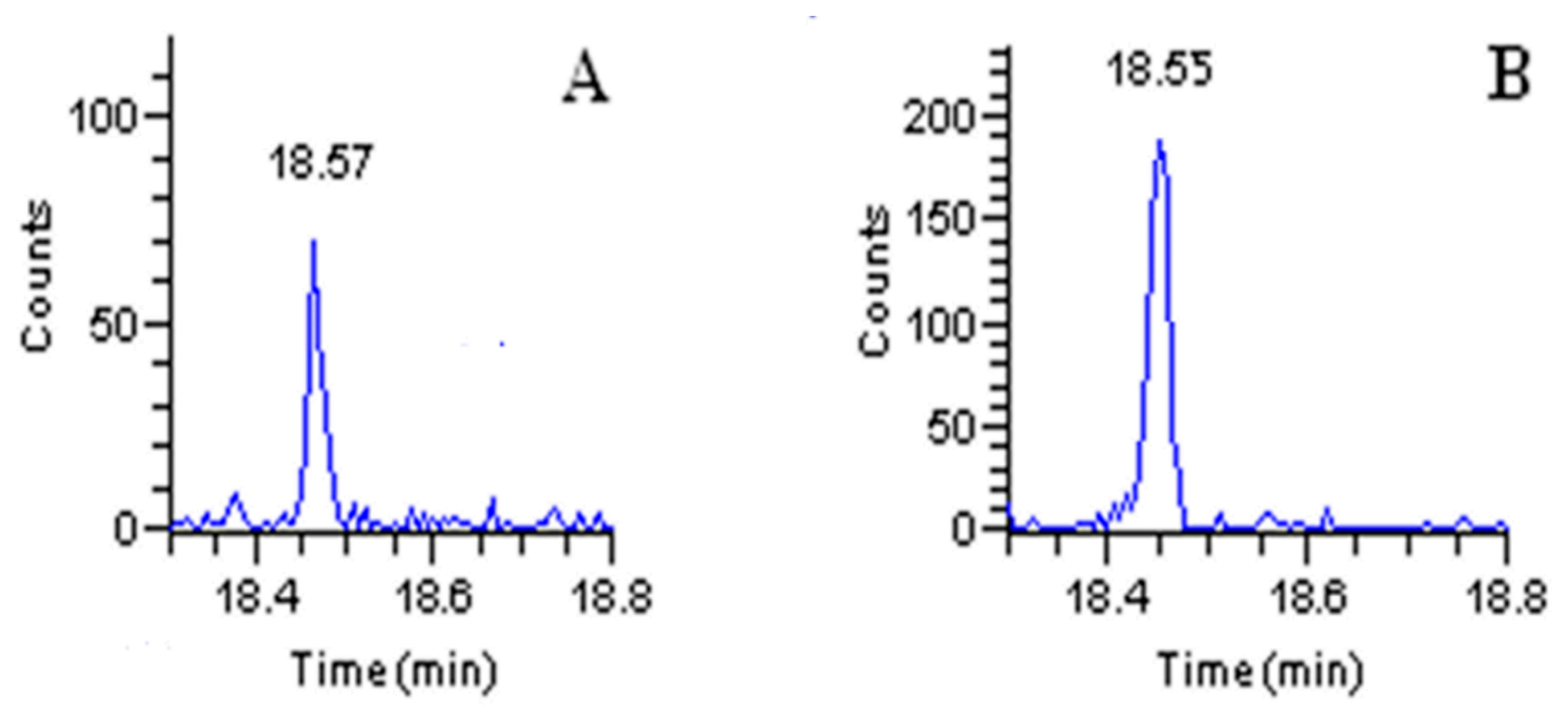
Detail of GC-MS chromatograms acquired in MS/MS mode for a naturally contaminated durum wheat semolina sample (**A**) and a type 00 soft wheat flour sample (**B**) containing diazinon at concentrations of 0.4 μg/kg and 1.0 μg/kg, respectively. The traces show the diazinon product ion at m/z 179 generated from the precursor ion at m/z 304.

**Table 1:** Linearity range, regression equation, correlation coefficient, LOD and LOQ for each analyte in SIM or MS/MS acquisition mode.

Pesticides	Acquisition Mode	Linearity Range * (ng/mL)	Equation (y = Area Count; x = ng/mL)	R^2^	LOD ** (ng/mL)	LOQ ** (ng/mL)
Ethoprophos	SIM	0.5–250	y = 8839x + 737	0.9994	0.20	0.70
	MS/MS	0.5–250	y = 2668x + 1093	0.9993	0.22	0.80
Dimethoate	SIM	1.0–250	y = 3583x + 271	0.9996	0.40	1.20
	MS/MS	1.5–250	y = 2587x + 164	0.9990	0.50	1.70
Terbufos	SIM	0.8–250	y = 20571x − 1376	0.9988	0.30	0.80
	MS/MS	0.1–250	y = 5508x − 201	0.9999	0.02	0.08
Diazinon	SIM	0.1–250	y = 64576x − 552	0.9995	0.05	0.14
	MS/MS	2.0–250	y = 1675x + 1142	0.9994	0.60	2.00
Aldrin	SIM	0.5–250	y = 44362x − 1103	0.9998	0.15	0.50
	MS/MS	0.3–250	y = 3004x − 187	0.9994	0.10	0.30
Chlorpyrifos	SIM	0.2–250	y = 6808x + 67	0.9998	0.07	0.20
	MS/MS	0.1–250	y = 3144x + 1592	0.9994	0.05	0.16
Bentazone	SIM	1.0–250	y = 2764x + 121	0.9995	0.40	1.30
	MS/MS	1.0–250	y = 1927x + 439	0.9991	0.40	1.30
Paclobutrazol	SIM	0.5–250	y = 2178x − 107	0.9996	0.15	0.50
	MS/MS	3.0–250	y = 1785x − 354	0.9990	1.0	3.30
Methoxychlor	SIM	0.5–250	y = 20080x + 1378	0.9996	0.25	0.80
	MS/MS	0.5–250	y = 1118x + 438	0.9995	0.25	0.80

Note: * The lowest calibration point corresponds to the lowest concentration meeting accuracy and precision criteria (≤20% RE and ≤20% RSD; see Section 4.5.1); ** LOD and LOQ were estimated mathematically using SD/slope; approach (see Section 4.5.2).

**Table 2:** Recoveries (%) and precision intra-day and inter-day.

Pesticide	Spike (n = 3)						Intra-Day RSD (n = 6)	Inter-Days RSD (n = 3, for 5 Days)
	5 μg/kg		20 μg/kg		50 μg/kg			
	After (%, ±SD)	Before (%, ±SD)	After (%, ±SD)	Before (%, ±SD)	After (%, ±SD)	Before (%, ±SD)		
Ethoprophos	106 ± 7	81 ± 14	100 ± 7	85 ± 15	101 ± 11	79 ± 12	11%	14%
Dimethoate	88 ± 4	13 ± 6	97 ± 3	30 ± 9	99 ± 9	25 ± 8	7%	15%
Terbufos	101 ± 8	97 ± 10	99 ± 7	95 ± 11	98 ± 7	96 ± 10	9%	13%
Diazinon	102 ±8	80 ± 10	112 ± 9	84 ± 10	110 ± 8	80 ± 11	9%	14%
Chlorpyrifos	79 ± 13	68 ± 23	80 ± 13	65 ± 22	79 ± 13	64 ± 24	15%	20%
Methoxychlor	97 ± 9	91 ± 11	107 ± 10	89 ± 15	101 ± 9	87 ± 15	12%	15%

Note: “After” = samples spiked after extraction (matrix effect + instrumental response); “Before” = samples spiked prior to extraction (overall process efficiency: extraction + matrix effect + instrumental response).

**Table 3:** Linearity range, regression equation, correlation coefficient, and estimated matrix-based limits of detection (LOD) and quantification (LOQ) for each analyte in composite blank matrix.

Pesticides	Linearity * (μg/kg)	Equation (y = Area Counts x = μg/kg)	R^2^	LOD ** (n = 6) (μg/kg)	LOQ ** (n = 6) (μg/kg)
Ethoprophos	0.2–200	y = 6968x + 230	0.9996	0.08	0.3
Dimethoate	5–200	y = 4660x − 1125	0.9978	1.5	5
Terbufos	0.3–200	y = 19,947x − 5677	0.9996	0.08	0.3
Diazinon	0.1–200	y = 262,060x − 35,872	0.9999	0.03	0.1
Chlorpyrifos	0.1–200	y = 46,281x + 2508	0.9998	0.03	0.1
Methoxychlor	0.2–200	y = 22,127x − 18,385	0.9998	0.1	0.3

Note: * The lowest calibration point corresponds to the lowest concentration meeting accuracy and precision criteria (≤20% RE and ≤20% RSD; see Section 4.5.1); ** LOD and LOQ were estimated mathematically using SD/slope; approach (see Section 4.5.2) and should not be interpreted as experimentally validated LODs or LOQs.

**Table 4:** Quantifier ion and confirmation ion(s) for each compound *.

Pesticide	t_R_ ± sd (min)	Selected Ion (m/z)	t_R_ Window (min)	Scan Event Time (min).
Ethoprophos	15.66 ± 0.06	97, **158,** 242	15.0–16.5	0.48
Dimethoate	17.84 ± 0.05	**87**, 93, 125	16.6–18.0	0.71
Terbufos	18.29 ± 0.06	97, **231,** 288	18.1–18.4	0.25
Diazinon	18.61 ± 0.06	137, **179**, 304	18.5–19.9	0.48
Aldrin	20.28 ± 0.07	**263**, 293, 315	20.0–20.3	0.34
Chlorpyrifos	20.48 ± 0.07	**197**, 258, 314	20.4–20.5	0.30
Bentazone	20.70 ± 0.06	119, **198,** 225	20.5–20.7	0.48
Paclobutrazol	21.65 ± 0.05	125, 167, **236**	20.8–23.0	0.71
Methoxychlor	25.02 ± 0.06	152, **227,** 344	23.1–25.8	0.25

Note: * The ion listed in **bold** was used as the quantifier ion, while the remaining ions served as qualifier ions. Analyte identification required the peak to fall within the specified retention-time window and the ion-ratio deviation between quantifier and qualifier ions to remain within the predefined acceptance limits. Supplementary Table S1 shows the library matching scores and relative intensity (%) of the major diagnostic ions used for analyte identification.

**Table 5:** Selected precursor ion, monitored product ion, and corresponding mass ranges.

Pesticide	Precursor Ion (m/z)	Product Ion (m/z)	Mass Range (m/z)
Ethoprophos	158	97	95–100
Dimethoate	125	93	90–95
Terbufos	231	97	95–100
Diazinon	304	179	176–182
Aldrin	263	193	190–196
Chlorpyrofos	197	169	166–172
Bentazone	198	119	116–122
Paclobutrazol	236	125	120–130
Methoxychlor	227	152	149–155

## Data Availability

The datasets generated and/or analyzed during the current study are available from the corresponding author upon reasonable request.

## Supplementary Materials

The following supporting information can be downloaded at https://www.mdpi.com/article/10.3390/molecules31203726/s1. Table S1: Library match scores and the main diagnostic ions used for analyte identification.
