# Supplementary material for: Determination of Selected Pesticide Residues in Wheat Flour by GC–MS
Source: Molecules. 2026 Oct 17;31(20):3726. doi: 10.3390/molecules31203726 (PMC13707824; doi:10.3390/molecules31203726)
Supplement: Supplementary file 1 [file molecules-31-03726-s001.zip › molecules-4597804-supplementary.pdf]

Supplementary Table S1: Library match scores and the main diagnostic ions used for analyte identification.

Analyte	Library Match Score (NIST)	Quantifier Ion (m/z, relative intensity)	Quantifier Ion	
			Ion 1 (m/z, relative intensity)	Ion 2 (m/z, relative intensity)
Ethoprophos	0.93	158 (100%)	97 (78%)	242 (38%)
Dimethoate	0.94	87 (100%)	93 (58%)	125 (44%)
Terbufos	0.92	231 (98%)	97 (37%)	288 (17%)
Diazinon	0.95	179 (100%)	137 (99%)	304 (55%)
Aldrin*	0.96	263 (87%)	293 (46%)	315 (18%)
Chlorpyrifos*	0.96	197 (92%)	314 (49%)	258 (28%)
Bentazone	0.91	198 (100%)	119 (99%)	225 (24%)
Paclobutrazol	0.93	236 (100%)	125 (74%)	167 (30%)
Methoxychlor	0.92	227 (100%)	152 (5%)	344 (4%)

Note: These values were obtained from the NIST mass spectral library searches performed during method development and correspond to the spectra acquired under the same GC–MS ion-trap conditions described in the manuscript. The table is provided to facilitate independent evaluation of spectral quality, given that Figure 1 cannot be enlarged due to production constraints.

- The ions monitored in MS/MS do not necessarily correspond to those observed under EI conditions, as collision-induced dissociation (CID) produces different and often more selective fragments. For Aldrin and Chlorpyrifos, the product ions 193 and 169 are the most stable and are widely reported in the literature for structural confirmation; therefore, these ions were selected for the transitions used in our method (DOI: 10.1109/ICBBE.2008.529; DOI: 10.1007/s12024-023-00720-4).
